# Adequate vitamin A liver stores estimated by the modified relative dose response test are positively associated with breastfeeding but not vitamin A supplementation in Senegalese urban children 9–23 months old: A comparative cross-sectional study

**DOI:** 10.1371/journal.pone.0246246

**Published:** 2021-01-29

**Authors:** Mane Hélène Faye, Marie-Madeleine A. Diémé, Nicole Idohou-Dossou, Abdou Badiane, Adama Diouf, Ndeye Magatte Ndiaye Ndome, Sherry A. Tanumihardjo

**Affiliations:** 1 Faculté des Sciences et Techniques, Laboratoire de Recherche en Nutrition et Alimentation Humaine, Département de Biologie Animale, Université Cheikh Anta Diop de Dakar, Dakar, Sénégal; 2 Région médicale de Dakar, Ministère de la Santé et de l’Action Sociale, Dakar, Sénégal; 3 Department of Nutritional Sciences, University of Wisconsin-Madison, Madison, Wisconsin, United States of America; University of Virginia, UNITED STATES

## Abstract

Vitamin A supplementation (VAS) in 6-59-month-old children is recommended but its sustainability is currently questioned. In Senegal, available data suggest that VAS should be maintained, but geographic and age-related specificities need to be addressed to better implement and target VAS programming. The objective of this comparative cross-sectional study, conducted in urban settings of Dakar, was to compare the vitamin A liver stores (VALS) assessed using the modified-relative dose response (MRDR) test between supplemented and non-supplemented 9–23 month-old children and to study their relationship with VAS. The supplemented group (n = 119) received VAS (either 100 000 UI or 200 000 UI) 2 to 6 months before evaluation while the non-supplemented group (n = 110) had not received VAS during the past 6 months. In addition to MRDR, serum retinol concentrations (SR), and biomarkers of subclinical inflammation were measured. Children’s health-related data and feeding patterns were collected. Mean MRDR values (VAS: 0.030 ± 0.017, non-VAS: 0.028 ± 0.016, P = 0.389) and inflammation-adjusted SR (VAS: 1.34 ± 0.37, non-VAS: 1.3 ± 0.35, P = 0.515) of children were adequate. Low prevalence of VALS (VAS: 5.2%, non-VAS: 5.4%) and inflammation-adjusted VAD (VAS: 2.6%, non-VAS: 0.9%) were detected despite high presence of infections and inflammation. Children were mostly still being breastfed (VAS: 85.7%, non-VAS: 77.3%) and complementary feeding indicators were similar in both groups. Only breastfeeding was associated with VALS and was found to reduce by 76% at least, the odds of VAD (adjusted OR = 0.24, 95% CI: 0.07–0.8, P = 0.020). Based on MRDR values, VAS was not related to improved VALS and SR as well as VAD reduction among these children with adequate VALS. Reinforcing breastfeeding advocacy and morbidity prevention/control are essential in this setting. Scaling-back VAS in this subpopulation should be examined regarding the risk of hypervitaminosis A after an evaluation of dietary vitamin A intake sufficiency and a more quantitative assessment of VALS.

## Introduction

Vitamin A deficiency (VAD) is a serious public health concern throughout the developing world, affecting 190 million preschool-age children and leading to many deleterious health consequences, including morbidity and mortality [1–3]. According to the most recent estimates, sub-Saharan Africa had the highest prevalence of VAD based on serum retinol concentrations (SR) lower than 0.7 μmol/L [4] and an analysis of existing VAD statistics in West Africa indicates a public health problem in children under five years [5–11].

Currently, strategies to prevent and control VAD and its consequences include dietary diversification, food fortification (either mandatory or voluntary), biofortification of staple foods and periodic high-dose vitamin A supplementation (VAS) [3]. In children, the latter remained the most widely implemented intervention delivering VA over the past few decades [3–12]. The World Health Organization (WHO) recommendation of high-dose VAS as a public health intervention to reduce morbidity and mortality in infants and children 6–59 months of age in settings where VAD is prevalent [13] was renewed in 2011 [14]. These guidelines are based on the reported effectiveness of VAS in reducing childhood mortality [15,16]. However, there is persistent controversy with reports from India [17] and Guinea Bissau [18] not finding any effect of VAS on young child mortality. Moreover, even though VAS was initially intended to protect against VAD by constituting liver stores to mobilize as needed, there is not sufficient evidence to support this assumption [13,16,19]. The effect of VAS on vitamin A (VA) status seems to be transient and consequently does not appear to sustainably reduce VAD [20]. Another raised concern is the safety of VAS in settings where multiple VA interventions coexist, increasing the risk of hypervitaminosis A [21] and its associated adverse effects [22]. Furthermore, sustainability of VAS programs has been recently questioned [23] given the burdensome financial and human resource costs and leadership provided mainly by external partners. Considering these issues, a policy shift from universal VAS towards more sustainable interventions or a systematic phase-out of VAS [24] or a more targeted strategy [25] have been suggested. Nevertheless, the Global Alliance for Vitamin A (GAVA) as well as others suggested that VAS should be pursued in countries or areas where VAD is still a public health problem until there is evidence of controlled VAD (i.e., VAD ≤ 10%) and children have a sustained and adequate VA intake from diet and other interventions [26–29].

In Senegal, large-scale VAS among 6 to 59 month-old children, has occurred twice a year since 1999 [30]. From 2009, mandatory VA fortification of refined cooking oil (VAFO) has been introduced. Even though the other interventions are implemented, their coverage is limited and their impact not sufficiently documented. According to the 2010 national survey, VAD among 12–59 month-old children (15.3%) was a moderate public health problem [11]. However, children under 2 years as well as those living in urban areas were less affected and their VA status was in the range of adequacy, especially those from Dakar, the Senegalese capital [11]. The under-five mortality rate also decreased significantly (from 121‰ to 56‰ between 2005 and 2017) [31,32] but prevalence of subclinical inflammation was still high (50.1%). Despite low VA intakes observed at the national level, using infant and young child feeding (IYCF) indicators, 6–23 month-old children in Dakar have the best dietary diversity (58.4% vs 25.3% nationally) and 76.6% of them consumed VA-rich foods regularly [32]. Coverage of VAS is low in this setting (46.6%), but consumption of fortified oil was found to cover more than a third of the household’s VA recommended safe intake [33]. This evidence suggests that even though VAS should be maintained in Senegal, it is also obvious that geography, age group and other specificities exist and should be addressed to better guide and implement the VAS program. To this end, more current data on VA status and its relationship to VAS are needed [34]. This study adds insight to this issue. The aim of this work was to compare vitamin A liver stores (VALS) assessed using the modified-relative dose response (MRDR) test between supplemented and non-supplemented 9–23 month old Senegalese children living in Dakar and to study the relationship with VAS. The MRDR test allows a semiquantitative estimate of liver VA and has been validated in animals as a function of liver VA stores [35,36]. It is less affected by the acute-phase response compared to SR [37,38] and better responds to interventions [39,40].

## Subjects and methods

### Study design, setting and population

A comparative cross-sectional study, involving two groups of healthy children who received VA supplements as retinyl palmitate [either 100 000 international units (IU) among 9–11 month old or 200 000 IU in 12–23 month old children] 2 to 6 months before the evaluation through the public health system as determined by records at the health posts (intervention group) and who had not received any VA supplements during the past 6 months (comparison group), was conducted in Dakar from September to November 2019. Exclusion criteria included clinical signs of severe infection and/or disease (diarrhea, dehydration, pneumonia), fever (temperature ≥ 37.5°C), severe anemia [Hemoglobin concentration (HB) < 7g / dL], malaria (positive test), severe acute malnutrition (WHZ <-3 z-scores) or severe obesity (WHZ > 3 z-scores). In addition, children of the VAS group should not have received more than one VA capsule during the 2 to 6 months prior to the study or any VA supplement in the past two months. The sample size determination was based on data from Tanumihardjo et al. [41] in Indonesian children (0.6 to 6.6 years) where the proportion of MRDR values ≥ 0.060 was 6% in a VAS plus deworming group compared to a control group (22%), four weeks after administration of supplements. Considering an *α* error of 5% and a power of 90%, the minimal sample size was estimated at 109 children per group. Using a non-response rate of 10%, 120 children per group were recruited. The study targeted the 4 administrative departments of Dakar and the sampling method consisted of random selection of one medical district per department and 2 health posts per district according to a probability proportional to size. Finally, children were identified from immunization records at 8 health posts. The study was approved by the ethical committees of the Senegalese Ministry of Health (Ref: SEN19/54) and the University Cheikh Anta Diop of Dakar (Ref: 0397/2019/CER/UCAD). Written and informed consent was obtained from the mothers or caregivers.

### Data collection procedures

According to the protocol, data collection was performed over 2 days. Mothers were first invited to confirm data on their children’s VAS using immunization cards and to carry out clinical examination, anthropometric measurements (i.e., weight and length), and anemia plus malaria testing. HB was measured using a HemoCue® 301+ portable device (HemoCue AB) and malaria testing was done using SD BIOLINE Malaria Antigen P.f/Pan® tests. Non-eligible children received appropriate clinical care from the health post. The second day, the MRDR test was performed in recruited children and a questionnaire was applied to respondents to collect mother’s and household characteristics (including consumption of fortified foods like oil and bouillon cubes), to evaluate children feeding practices (i.e., breastfeeding and complementary feeding practices), and to record health-related information (occurrence of diarrhea, cough/ breathing troubles, fever, skin rash). 3,4-Didehydroretinyl acetate was synthesized using published procedures, purified on 8% deactivated alumina, and dissolved directly into soybean oil for storage at -80°C until hand-carried to Senegal. The MRDR test consists of administering a standard oral dose for children <2 years old of 3.5 μmol 3,4-didehydroretinyl acetate diluted in 480 μL soybean oil to each child using a Gilson positive displacement micropipette or 1 mL graduated syringe. Immediately after dose administration, children received either a peanut butter spread with bread or 480 μL of unfortified vegetable oil if they were not able to eat the snack to maximize absorption. Finally, 4 to 6 hours after dosing, a venous blood sample was collected and centrifuged at 3000 rpm for 10 minutes at ambient temperature. Serum handling was done away from dust, under minimal light, and samples were wrapped with aluminum foil to protect light-sensitive compounds from degradation. Serum samples were separated into cryovials and placed in a cooler while in the field and stored at -80°C at the laboratory until analysis. Serum concentrations of 3,4-didehydroretinol, retinol, C-reactive protein (CRP) and α1-acid-glycoprotein (AGP) were measured.

### Biochemical measurement procedures

The measurement of 3,4-didehydroretinol and retinol was performed by high performance liquid chromatography using a Thermo Scientific™ system consisting of a P4000 pump, an AS3000 autosampler, an UV8000 detector, and a SN4000 module. Once the serum samples were thawed, a 400 μL aliquot was treated with 250 μL ethanol (to denature proteins) and extracted twice with 300 μL hexane in a glass test tube. Hexane layers were combined, evaporated under nitrogen and the dry extract was dissolved in 80 μl of 75:25 (v:v) methanol: dichloroethane to dissolve the lipid phase. Then, 40 μL of the resuspended volume was injected into a Waters Resolve^TM^ C18 (5 μm 90 Å 3.9×150 mm), protected with a guard column. The wavelength was set at 350 nm to optimize 3,4-didehydroretinol detection. The flow rate was 1 mL/min and the mobile phase was 89:11 (v:v) methanol: water (with 0.05% trimethylamine as stabilizer). To determine extraction efficiencies, retinol acetate was used as an internal standard. The external standard of 3,4-didehydroretinol was prepared by saponifying the acetate form with alcoholic potassium hydroxide and purifying by HPLC. Commercial retinol (Sigma-Aldrich) was also purified and used as an external standard. Standard curves were constructed to quantify 3,4-didehydroretinol and retinol in the serum samples. All procedures were performed under yellow light to minimize VA destruction and isomerization.

Serum CRP and AGP concentrations were quantified by immuno-turbidimetry using a Biosystems A15 automatic analyzer (Biosystems S.A.) with Biosystems kit reagents.

### Case definitions

Low/inadequate VALS were defined as a MRDR ratio ≥ 0.060 [42,43] and a cutoff for SR ≤ 0.7 μmol/L was used to define VAD [38]. Inflammation status was defined as CRP > 5 mg/L and/or AGP > 1 g/L [44]. Four groups were defined based on CRP and AGP levels: (a) reference (normal CRP and AGP), (b) incubation (raised CRP and normal AGP); (c) early convalescence (raised CRP and AGP), and (d) late convalescence (normal CRP and raised AGP). Children’s weight-for-length (WLZ) and length-for-age (LAZ) z-scores were calculated according to the WHO growth standard references [45]. Moderate wasting and moderate stunting were defined as -3 z-scores ≤ WLZ < -2 z-scores and -3 z-scores ≤ LAZ < -2 z-scores, respectively. HB is expressed in g/dL and moderate to mild anemia were defined as 7 g/dL ≤ HB < 11 g/dL [46].

### Assessment of complementary feeding practices

Complementary feeding practices were assessed using IYCF indicators per WHO methodology with a 24-hour recall period [47]. Dietary diversity score (DDS) was calculated based on the number of food groups consumed among 7 during the preceding 24 hours. These 7 food groups are: (1) grains, roots and tubers, (2) legumes and nuts, (3) dairy products (milk, yogurt, cheese), (4) flesh foods (meat, fish, poultry and liver/organ meats), (5) eggs, (6) vitamin-A rich fruits and vegetables and (7) other fruits and vegetables. Minimum dietary diversity (MDD), an indicator reflective of children’s diet quality, is defined as the consumption of foods from at least 4 out of these 7 food groups (DDS ≥ 4). Minimum meal frequency (MMF) was defined as the consumption of solid, semi-solid, or soft foods (but also including milk feeds for non-breastfed children) the minimum number of times or more. The minimum number is 3 times for breastfed children 9–23 months and 4 times for non-breastfed children 6–23 months. This indicator is intended as a proxy for energy intake from foods other than breast milk. Minimum acceptable diet (MAD), which is a composite indicator, was defined as at least the MDD and MMF during the previous day in breastfed children and at least 2 milk feeds, the MDD not including milk feeds and the MMF during the previous day for non-breastfed children.

### Statistical analysis

Data entry and quality control were done using Epi info^TM^ 7.2.3.1, Epi info^TM^ 3.5.1 (Centers for Disease Control and Prevention) and Microsoft Excel 2016 (Microsoft Corporation). Statistical analysis was performed using STATA/SE 12.0 (STATA Corporation). Descriptive analysis tabulated the characteristics of the study population. Categorical variables were expressed as percentages, and continuous variables were expressed as mean ± SD for normally distributed variables and median with interquartile range for skewed values. Two-tailed Student’s *t*-test, Paired *t*-test or one-way analysis of variance (ANOVA) followed by Bonferroni's post-hoc comparison tests were used to compare means while the Wilcoxon rank-sum test was used to compare medians between groups. Pearson's chi-squared test, Fisher’s exact test or McNemar’s chi-squared test were used to compare percentages.

The relationship between SR and MRDR values, as well as between each of these two variables and inflammation biomarkers was studied using Pearson’s correlation coefficient (r). For this purpose, CRP values were log-transformed as they were not normally distributed. MRDR and SR values were adjusted using linear prediction from a regression model including age, weight, length and supplementation groups as independent variables. However, in absence of differences between adjusted and non-adjusted values of both MRDR and SR, cutoffs for VAD were applied to unadjusted values of these variables. SR was then adjusted for subclinical inflammation using the regression correction approach developed by the BRINDA project as previously described [48,49] and inflammation-adjusted prevalence of VAD was derived from those values. Briefly, adjusted SR values were obtained by subtracting the influence of CRP and AGP as follows: Retinol_**adjusted**_ = Retinol_**unadjusted**_−β1 (CRP_**obs**_—CRP_**ref**_)–β2 (AGP_**obs**_—AGP_**ref**_). In this equation, β1 and β2 are regression coefficients of CRP and AGP, respectively, obs is the individual observed value and ref is the reference value. Retinol, CRP_**obs**_, CRP_**ref**_, AGP_**obs**_ and AGP_**ref**_ are on natural logarithm scale. Internal reference values from our dataset (maximum value of the lowest CRP or AGP decile or 10th percentile obtained) were used. Unlogged CRP_**ref**_ was 0.3 mg/L while AGP_**ref**_ was 0.72 g/L. Adjustments were only applied to individuals with either CRP concentrations ˃ CRP_**ref**_, AGP concentrations ˃ AGP_**ref**_ or both. SR-adjusted values were back-transformed before applying cutoffs. Univariate and multivariate logistic regression controlling with covariates was used to identify associated factors of VALS in children. For all statistical analyses, a P value < 0.05 was used for significance.

Using household characteristics (household occupancy, drinking water source, type of toilet facilities, type of fuel used for cooking) and ownership of durable goods (household equipment and livestock), a socio-economic status (SES) index was generated using principal component analysis [50] where the first component explained 14.3% of the variance. Based on each household’s index score, a household SES indicator consisting of 3 categories (lowest, intermediate and highest) was obtained.

## Results

### Characteristics of the study population

**Fig 1:**
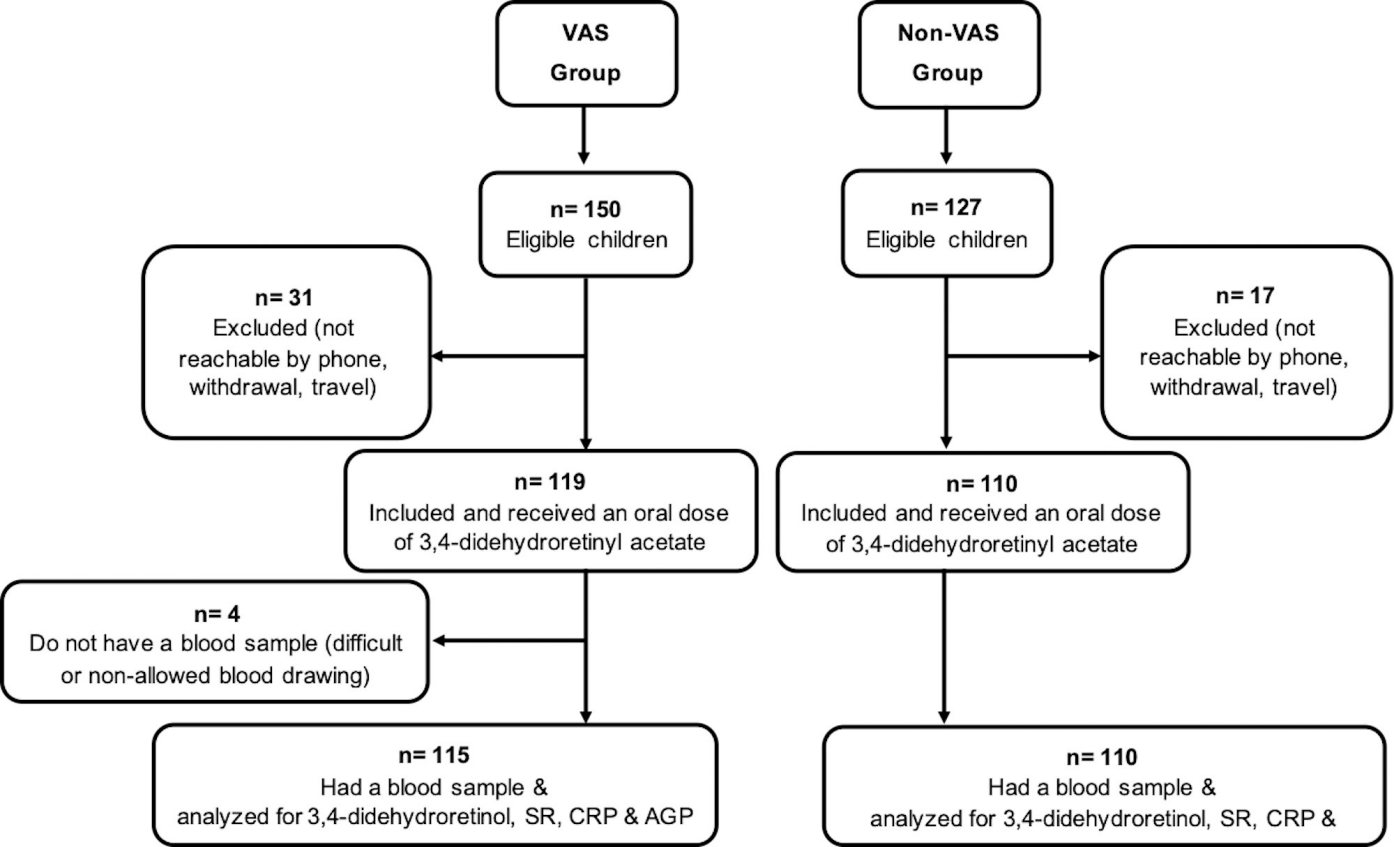
Flow chart of children 9 to 23 months old recruited for the study, Senegal, 2019. VAS group: Vitamin A supplemented group; non-VAS group: Non-vitamin A supplemented group; SR: Serum retinol; CRP: C-reactive protein; AGP: Alpha1-acid glycoprotein.

Among the eligible children, a total of 119 in the VAS group and 110 in the non-VAS group were recruited and received an oral dose of 3,4-didehydroretinyl acetate. However, 4 children in the VAS group did not have blood samples resulting in 115 children in the VAS group and 110 children of the non-VAS group assessed for 3,4-didehydroretinol, SR, CRP and AGP. The study flow chart is presented in Fig 1.

The analysis of the children’s characteristics (Table 1) shows a comparable distribution according to gender in the two groups. However, children in the non-VAS group were older (14.6 months [12.6–19.5] vs 11.9 months [11–14.7], P < 0.001), heavier (9.4 ± 1.4 vs 8.9 ± 1.0, P = 0.0012) and taller (76.9 ± 4.9 vs 74.5 ± 4.0, P < 0.001) than the VAS children. The non-VAS group presented significantly higher proportion of stunting (11.8% vs 4.2%, P = 0,032), but this difference disappeared when a case-control comparison was performed by matching a VAS child and a non-VAS child by age and sex (VAS group: 3.5% [95% CI: 0.004–0.12] vs non-VAS group: 12.3% [95% CI: 0.05–0.24], P = 0.180).

**Table 1:** Characteristics of the children^1^.

Characteristics	VAS group (n = 119)	Non-VAS group (n = 110)	P value^9^
Sex			
Girls	58 (48.7)	56 (50.9)	0.743
Boys	61 (51.3)	54 (49.1)	
Age group, months			
09–11	62 (52.1)	21 (19.1)	<0.001
12–17	40 (33.6)	55 (50.0)	
18–23	17 (14.3)	34 (30.9)	
Nutritional status			
WLZ	-0.48 ± 1.02	-0.44 ± 1.03	0.730
WLZ, matched analysis^2^	-0.41 ± 1.09	-0.39 ± 1.13	0.918
Moderate wasting^3^	9 (7.6)	9 (8.2)	0.862
Moderate wasting^3^, matched analysis^2^	3 (5.3)	5 (8.8)	0.726
LAZ	-0.36 ± 1.05	-0.60 ± 1.14	0.097
LAZ, matched analysis^2^	-0.44 ± 1.13	-0.59 ± 1.17	0.482
Moderate stunting^4^	5 (4.2)	13 (11.8)	0.032
Moderate stunting^4^, matched analysis^2^	2 (3.5)	7 (12.3)	0.180
Moderate to mild Anemia^5^	59 (49.6)	59 (53.6)	0.539
Illness^6^	98 (82.3)	96 (87.3)	0.265
Fever^7^	70 (58.8)	64 (58.2)	0.865
Diarrhea^7^	57 (47.9)	48 (43.6)	0.481
Cough/ breathing trouble^7^	33 (27.7)	24 (21.8)	0.268
Skin rash^7^	42 (35.3)	46 (41.8)	0.304
Deworming^8^	52 (43.7)	50 (45.4)	0.934

VAS group: Vitamin A supplemented group, non-VAS group: Non-vitamin A supplemented group.

^1^Values are mean ± SD or n (%).

^2^Matched analysis: n = 57 pairs.

^3^–3 z-scores ≤ WLZ < -2 z-scores.

^4^–3 z-scores ≤ LAZ < -2 z-scores.

^5^7 g/dL≤ HB < 11 g/dL.

^6^Children who suffered from at least one symptom of infection (fever, diarrhea, cough/breathing trouble and/or skin rash) within the two weeks prior to the study.

^7^Reported within the past 15 days.

^8^Reported within the past 6 months.

^9^P value of Pearson's chi-squared test, Fisher’s exact test or McNemar’s chi-squared test for proportions; Student *t* test for means.

In regards to the reported occurrence of illnesses, 82.3% (n = 98) and 87.3% (n = 96) of children suffered from at least one symptom of infection (fever, diarrhea, cough/breathing trouble and/or skin rash) within the past 15 days before the study in the supplemented and the non-supplemented groups, respectively. Taken separately, the distribution of these diseases was analogous in the two groups. Almost 1 out of 2 children benefited from deworming within the past 6 months in each group.

There was no difference regarding mothers’ characteristics and households’ socioeconomic status between the two groups (Table 2). The mean age of mothers was 29.9 ± 6.8 years in the VAS group vs 28.4 ± 6.5 years in the non-VAS group (P = 0.114). The median number of household members was 10 [6–15] and 9 [5–13] (P = 0.157), and children under five were 2 [1–3] (P = 0.071) in the two groups, respectively. Consumption of VAFO was high in our study population (94.1% vs 95.4% in VAS and non-VAS group, respectively [P = 1.000]). Consumption of VA-fortified bouillon cubes was also found in 22.7% and 32.7% of households in the two groups, respectively.

**Table 2:** Characteristics of mothers and households^1^.

Characteristics	VAS group (n^2^ = 119)	Non-VAS group (n^2^ = 110)	P value^3^
**Mothers**			
Marital status			
Married	112 (94.1)	102 (92.7)	0.527
Ethnicity			
Wolof/Lébou	34 (28.6)	32 (29.1)	0.573
Toucouleur/Pulaar	30 (25.2)	34 (30.9)	
Sereer	29 (24.4)	18 (16.4)	
Manding/Soce/Bambara	10 (8.4)	9 (8.2)	
Diola	6 (5.0)	4 (3.6)	
Other	8 (6.7)	12 (10.9)	
Education			
No formal	19 (16.0)	20 (18.2)	0.946
Formal	76 (63.9)	72 (65.4)	
Koranic	15 (12.6)	14 (12.7)	
Vocational	3 (2.5)	1 (0.9)	
Other	2 (1.7)	2 (1.8)	
Occupation			
Unemployed	63 (52.9)	58 (52.7)	0.946
Employed	51 (42.9)	50 (45.4)	
Volunteer	1 (0.84)	1 (0.91)	
Parity			
Primiparous	32 (26.9)	28 (25.4)	0.777
Multiparous	85 (71.4)	81 (73.6)	
**Households**			
SES			
Lowest	34 (28.6)	42 (38.2)	0.202
Middle	45 (37.8)	31 (28.2)	
Higher	39 (32.8)	35 (31.8)	
Consumption of fortified foods/ food additives			
Vitamin A fortified oil	112 (94.1)	105 (95.4)	1.000
Vitamin A fortified bouillon cubes	27 (22.7)	36 (32.7)	0.157

VAS group: Vitamin A supplemented group, non-VAS group: Non-vitamin A supplemented group; SES: Socio-economic status.

^1^Values are n (%).

^2^The sum of subgroups may not equal the total because of missing data.

^3^P value of Pearson's chi-squared test or Fisher’s exact test based on valid entries.

### Breastfeeding and complementary feeding indicators

Breastfeeding and complementary feeding indicators (Table 3) showed similar patterns in the VAS and non-VAS groups. Over 3/4 of the children were still being breastfed in both groups. Among weaned children, the median duration of breastfeeding was 18.5 months [16.5–19.5] and 18 months [16–20] in the VAS and non-VAS groups, respectively. The overall mean DDS in each group was below the cutoff of 4 food groups and MDD was met by 45.4% in the VAS group and 54.5% in the non-VAS group. However, when children were separated according to whether they were breastfed or not, non-breastfed children had an adequate mean DDS and most of them had MDD. Almost sixty-four percent (63.9%) and 72.7% of VAS and non-VAS children met MMF, respectively. However, only 1/3 of the children had MAD in the two groups and none of the non-breastfed children met MAD.

**Table 3:** Level of selected IYCF indicators^1^.

IYCF indicators	VAS group (n^2^ = 119)	Non-VAS group (n^2^ = 110)	P value^3^
Currently breastfeeding			
Yes	102 (85.7)	85 (77.3)	0.095
No	16 (13.4)	24 (21.8)	
Dietary diversity			
DDS^4^, all	3.39 ± 1.63	3.66 ± 1.35	0.183
MDD^5^, all	54 (45.4)	60 (54.5)	0.182
DDS, breastfed children^6^	3.22 ± 1.58	3.41 ± 1.28	0.365
MDD, breastfed children	42 (41.2)	40 (47.1)	0.454
DDS, non-breastfed children^7^	4.50 ± 1.55	4.54 ± 1.25	0.926
MDD, non-breastfed children	12 (75.0)	20 (83.3)	0.519
Meal frequency			
MMF^8^^,^^9^, all	76 (63.9)	80 (72.7)	0.170
MMF, breastfed children	63 (61.8)	61 (71.8)	0.176
MMF, non-breastfed children	13 (81.2)	19 (79.2)	0.872
Minimum acceptable diet^10^			
MAD, all	39 (32.8)	36 (32.7)	0.961
MAD, breastfed children	39 (38.2)	36 (42.3)	0.605
MAD, non-breastfed children	0 (0)	0 (0)	-

IYCF: Infant and young child feeding; VAS group: Vitamin A supplemented group, non-VAS group: Non-vitamin A supplemented group; DDS: Dietary diversity score; MDD: Minimum dietary diversity; MF: Meal frequency; MMF: Minimum meal frequency; MAD: Minimum acceptable diet.

^1^Values are mean ± SD or n (%).

^2^The sum of subgroups may not equal the total because of missing data.

^3^P value of Pearson's chi-squared test or Student *t* test based on valid entries.

^4^DDS is defined as number of food groups consumed over 7 during the preceding 24 hours. These 7 foods groups are: (1) grains, roots and tubers, (2) legumes and nuts, (3) dairy products (milk, yogurt, cheese), (4) flesh foods (meat, fish, poultry and liver/organ meats), (5) eggs, (6) vitamin-A rich fruits and vegetables and (7) other fruits and vegetables.

^5^DDS ≥ 4.

^6^VAS group: n = 101, non-VAS group: n = 85.

^7^VAS group: n = 16, non-VAS group: n = 24.

^8^MF is defined as consumption frequency of solid, semi-solid, or soft foods (but also including milk feeds for non-breastfed children) during the preceding 24 hours.

^9^MMF is defined as MF ≥ 3 times for breastfed children 9–23 months and MF ≥ 4 times for non-breastfed children 6–23 months.

^10^For breastfed children: MDD and MMF during the previous day; for non-breastfed children: MDD (not including milk feeds) and MMF and at least 2 milk feedings.

### Subclinical inflammation

Median values of CRP and mean values of AGP were comparable in the two groups (Table 4). Subclinical inflammation was found in almost 3 out of 4 children in the 2 groups with a higher proportion of children in early convalescence.

**Table 4:** Comparison of indicators of subclinical inflammation between VAS and non-VAS children^1^.

	VAS group (n = 115)	Non-VAS group (n = 110)	P value
CRP in mg/L, median	4.9 [2.1–15.7]	4.5 [2.3–15.4]	0.908
AGP in g/L	1.24 ± 0.69	1.18 ± 0.5	0.414
Inflammation			
Yes^2^	84 (73.0)	80 (72.7)	0.957
No	31 (27.0)	30 (27.3)	
Stage of inflammation^3^			
No inflammation	31 (27.0)	30 (27.3)	0,491
Incubation	11 (9.6)	17 (15.4)	
Early convalescence	46 (40.0)	36 (32.7)	
Late convalescence	27 (23.5)	27 (24.5)	

VAS group: Vitamin A supplemented group, non-VAS group: Non-vitamin A supplemented group.

^1^Values are n (%), mean ± SD or median [IQR].

^2^Any inflammation: CRP> 5 mg/L and/ or AGP> 1 g/L.

^3^Stage of inflammation: incubation (CRP > 5 mg/L and AGP ≤ 1 g/L); early convalescence (CRP> 5 mg/L and AGP> 1g/L); late convalescence (AGP>1 g/L and CRP≤ 5 mg/L).

### Vitamin A liver stores

Regarding VA status indicators of children, results are presented in Table 5. The MRDR mean ratio (DR:R) was 0.030 ± 0.017 in the VAS children and 0.028 ± 0.016 among their non-VAS counterparts (P = 0.389). After adjustment for age, weight and length, the mean MRDR ratios were 0.028 ± 0.016 vs 0.029 ± 0.016 for the supplemented and the non-supplemented children, respectively (P = 0.565). However, adjusted MRDR values did not differ from the unadjusted one in the VAS group (0.028 ± 0.016 vs 0.030 ± 0.017, P = 0.466) and the non-VAS group (0.029 ± 0.016 vs 0.028 ± 0.016, P = 0.477). Therefore, prevalence of inadequate VALS was derived from unadjusted values.

**Table 5:** Comparison of indicators of vitamin A status between VAS and non-VAS children^1^.

	VAS group (n = 115)	Non-VAS group (n = 110)	P value
DR:R ratio (unadjusted)	0.030 ± 0.017	0.028 ± 0.016	0.389
DR:R ≥ 0.060 (low liver stores)	6 (5.2)	6 (5.4)	0.937
Unadjusted SR, μmol/L	1.21 ± 0.34	1.18 ± 0.32	0.513
Inflammation-adjusted SR, μmol/L	1.34 ± 0.37	1.3 ± 0.35	0.515
Unadjusted VAD^2^	6 (5.2)	4 (3.6)	0.565
Inflammation-adjusted VAD^2^	3 (2.6)	1 (0.9)	0.335

VAS group: Vitamin A supplemented group, non-VAS group: Non-vitamin A supplemented group, DR: 3,4-didehydroretinol, R: Retinol, SR: Serum retinol.

^1^Values are n (%) or mean ± SD.

^2^VAD: Serum retinol ≤ 0.7 μmol/L).

Among non-VAS children, the mean MRDR value of those who received at least one VA supplement since birth was similar to that of those who never received any VA supplements (0.026 ± 0.017 vs 0.031 ± 0.015, P = 0.232). No correlation was noticed between the MRDR mean ratio and serum CRP (r = -0.0408, P = 0.572), contrary to AGP (r = 0.1351, P = 0.043). However, this correlation with AGP disappeared after exclusion of 3 outliers (r = 0.078, P = 0.25). Using a cutoff of MRDR ≥ 0.060, only 5.2% (n = 6) and 5.4% (n = 6) of children had inadequate VALS in the VAS and the non-VAS groups, respectively, with no difference between groups (P = 0.937).

### Serum retinol concentrations

The mean SR was 1.21 ± 0.34 μmol/L in VAS children and 1.18 ± 0.32 μmol/L in non-VAS children (P = 0.513) (Table 5). Adjustment for age, weight and length did not significantly affect the mean adjusted compared to non-adjusted SR values in the VAS group (1.21 ± 0.34 μmol/L vs 1.21 ± 0.34 μmol/L, P = 0.992) and in the non-VAS group (1.18 ± 0.34 μmol/L vs 1.18 ± 0.32 μmol/L, P = 0.947). Due to this, the prevalence of VAS based on unadjusted values of SR is presented.

Unadjusted VAD was 5.2% (n = 6) and 3.6% (n = 4) in VAS and non-VAS children, respectively, with no significant difference between groups (P = 0.565). Overall, SR were negatively and significantly correlated with CRP and AGP (CRP: r = -0.1573, P = 0.029; AGP: r = -0.1849, P = 0.006). However, when the analysis was performed by VAS status, SR was only significantly correlated with CRP in the VAS group (CRP: r = -0.2108, P = 0.037; AGP: r = -0.1464, P = 0.12) and with AGP in the non-VAS group (CRP: r = -0.0984, P = 0.34; AGP: r = -0.2510, P = 0.008). After adjustment using the BRINDA regression methodology, the mean adjusted SR of children increased significantly in each of the groups (VAS: 1.21 ± 0.34 μmol/L vs 1.34 ± 0.37 μmol/L, P < 0.001; non-VAS: 1.18 ± 0.32 μmol/L vs 1.3 ± 0.35 μmol/L, P < 0.001). However, inflammation-adjusted SR did not differ between groups. Overall, the median adjusted SR was 1.3 μmol/L [1.1–1.5] and 11.5% of children were above 1.6 μmol/L. The inflammation-adjusted VAD was 2.6% (n = 3) and 0.9% (n = 1) in VAS and non-VAS children respectively, with no significant difference between groups (P = 0.335).

### Relationship between indicators of vitamin A status

A negative and significant correlation was found between unadjusted MRDR and inflammation-adjusted SR (r = -0.2330, P < 0.001) and the same scheme was observed in each of the 2 groups (VAS group: r = -0.2314, P = 0.013; non-VAS group: r = -0.2417, P = 0.011).

Overall, based on MRDR values, inflammation-adjusted SR and cutoffs defining deficiency, 93.3% of the children (n = 210) were true negatives (i.e., classified non-VAD by the two biomarkers: MRDR-, SR-) and only one child was a true positive (i.e., classified VAD by the two biomarkers: MRDR+, SR+). False negatives (MRDR+, SR-) and false positives (MRDR-, SR+) were 4.9% (n = 11) and 1.3% (n = 3), respectively. Based on these figures and compared with the MRDR test, the sensitivity of inflammation-adjusted SR to detect VAD was low (8.3%) and its specificity, positive predictive value and negative predictive value were 98.6%, 25% and 95%, respectively.

### Factors associated with vitamin A liver stores

Regarding the low number of cases with poor VALS in this study population, children were pooled to identify the factors associated with VALS. In univariate logistic regression analysis, VALS were not associated with VAS (OR = 1.05, 95% CI: 0.3–3.3, P = 0.937) even after controlling for covariates (i.e., age, height and length) (OR = 0.98, 95% CI: 0.3–3.4, P = 0.972). Using a multivariate regression of relevant significant variables in bivariate analysis, breastfeeding was found to reduce by 76% the odds of VAD (adjusted OR = 0.24, 95% CI: 0.07–0.8, P = 0.020). When performing additional adjustments using covariates (i.e., age, height and length), the percent reduction of the risk of inadequate VALS increased to 89% (adjusted OR = 0.11, 95% CI: 0.02–0.72, P = 0.021).

In the VAS group, VALS were not associated with the duration since last supplementation in children (OR = 1.57, 95% CI: 0.7–3.4, P = 0.246). Nevertheless, all children in this group who had inadequate VALS and/or deficient SR (n = 8) had received their supplements ≥ 4 months before.

## Discussion

VAS is recommended by WHO in areas at risk for deficiency but is currently questioned for many reasons among different settings [51,52]. Thus, contextual investigations at the country or sub-country level are needed to better address and target its programming. The purpose of this study was to compare VALS between supplemented and non-supplemented 9–23 month-old children of urban areas of Dakar and to study the relationship between VALS and VAS. The MRDR revealed a very low prevalence of VAD, which agreed with inflammation adjusted SR.

### Difference in age between VAS and non-VAS children

In this study, a difference in age was observed between the two groups and might be related to the routine delivery mode of VA supplements during immunizations at 9 months. This impacts the coverage of VAS being high in 6–11 months compared to those over 12 months. This finding corroborates previous observations [30] and the bivariate association between children’s age group and the reception of VAS observed in sub-Saharan African countries [53]. This difference in age between the two groups may also explain the difference in the proportion of stunting that disappeared when the comparison was performed after matching VAS and non-VAS children by age and sex.

### VAS is not associated with improved VA liver stores or a reduction of VAD

The mean MRDR ratio observed in the two groups is approximately 0.030, which depicts adequate VALS among these children. These values are lower (i.e., better VA liver reserves) than figures previously reported in Senegalese 6 month-old infants but similar to those reported in Ghanaian children aged 7 to 9 months [54,55]. No difference was observed between the supplemented and the non-supplemented group regarding mean MRDR values. The analysis of no difference suggests three possibilities: 1) that VAS did not improve VA status 2 to 6 months after its administration among healthy 9 to 23 month old children living in urban areas of Dakar compared to non-VAS children, which could be linked with their underlying adequate VA status; 2) that VALS were improved by VAS but deteriorated by this time in whatever elevation was initially obtained, and 3) that the MRDR test, which is not quantitative, may not have detected the difference between the groups due to the duration between VAS and VALS’s assessment. Indeed, many studies obtained an improvement following VAS in young children using the MRDR test. Tanumihardjo et al. found an improvement of the mean MRDR ratio from 0.054 ± 0.038 to 0.029 ± 0.018 (P<0.0001) 4 weeks after a 210 μmol (200 000 IU) VAS plus deworming in Indonesian children 0.6 to 6.6 years infected with *Ascaris Lumbricoides* [41]. In another study, the same authors observed that the MRDR ratio of a group of children within one-month post-VAS was better compared to that of children who were administered VAS ≥ 4 months before [56]. This improvement was likely due to the underlying VAD in many of these Indonesian children reflected in the high mean MRDR value at baseline and the fact that one month is a shorter time interval. In the study by Ayah et al., a significant decrease of MRDR ratios (-0.012, 95% CI: -0.019, -0.005; P = 0.001) was observed among VA supplemented Kenyan infants (100 000 IU) compared to those who were not, 3 months after administration [57]. More recently, reports from South African preschool children using the retinol isotope dilution technique (RID) showed a significant shift in total liver vitamin A reserves (TLRs) from 1.13 ± 0.43 to 1.29 ± 0.4 μmol/g (P < 0.001) before and 4 weeks after high-dose VA [58]. However, based on MRDR values, it would seem that even though VALS might be improved following VAS, this could not be maintained to 6 months post-administration, especially in children prone to infections like in our study population. The lack of difference in SR between the VAS and the non-VAS groups in these Senegalese children and the fact that all children with inadequate liver stores and/or VAD in the VAS group had received their supplements ≥ 4 months before are in line with conclusions of a transient effect of VAS on VA status as previously observed [20]. Moreover, our results showed a similar prevalence of low liver stores and VAD (SR ≤ 0.7 μmol/L) in both groups, suggesting no benefit of VAS in reducing VAD [16,24,59]. This hypothesis is in accordance with the observed lack of association between VALS and VAS and may be linked with an adequate baseline VA status of the children.

### Low prevalence of VAD due to multiple strategies

The prevalence of low liver stores found in our study population is similar to that of 6.8% reported by Kafwembe et al. in under five Zambian children from a community with both VAS and VA fortification of sugar and that of 6.9% observed by Newton et al. among non-supplemented 7–9 month-old infants in Ghana [55,60]. However, this is drastically lower than the prevalence of 73.5% previously observed among 6 month-old infants living in Dakar [54], suggesting an improvement of VA status beyond 6 months of age and enough dietary intake to build VA reserves. Accordingly, mean SR observed in this study (both among VAS and non-VAS) were higher than the values reported earlier for 12–23 month-old children from urban Dakar [11,54]. Moreover, the overall inflammation-adjusted prevalence of VAD (1.8%) was lower than the 6.9% found earlier [11]. This improvement might be due to synergistic contributions from diverse national strategies.

The analysis of complementary feeding indicators, both overall and by VAS status, revealed that MMF almost doubled proportions reported from DHS 2017 even though MDD, the indicator of diet quality, is similar to previously reported figures [32]. However, although MAD is still low in both groups, it has also doubled from 2017 DHS data (12.3%). These observations could reflect improvements in children’s dietary VA intakes despite these indicators still needing enhancement. However, quantitative proof of adequate VA intakes is still needed.

Furthermore, it should be emphasized that a large-scale mandatory VA fortification program of cooking oil (VAFO) was implemented since 10 years [61]. This intervention probably contributed positively to daily requirements for children while improving breastmilk VA concentrations in lactating mothers as found in Indonesia, but not in Cameroon due to the low coverage of VAFO [62,63]. VAFO was also found to improve VA status in Filipino children [59]. This hypothesis is plausible given the coverage of VAFO found in this study (over 95%) and that of 73% among women of reproductive age at the national level [61]. Also, high intakes of VAFO cover at least one-third of recommended safe intakes (RSI) of urban households in Dakar [33]. Assuming a daily median consumption of 12 g/day of fortified oil (11 to 24 mcg RE/kg), between 33% to 72% of daily needs could be met by 12–59 month-old children in urban areas of Senegal [64,65]. This study is timely and the impact of VAFO should be further evaluated to confirm these assumptions. Consumption of VA-fortified bouillon cubes was found in at least 25% of child households also contributing to VA intakes, given the high consumption of these kinds of additives (91.2%) recently reported in this setting [66]. However, to our knowledge, there are no published data available to support this finding.

Moreover, the adequacy of children’s liver reserves was associated with breastfeeding, the latter found to reduce by 76% at least, the odds of VAD (as DR:R ≥0.060). The relationship between breastfeeding and VAD has been reported in a case-control study involving a sample of 6–35 month-old children treated for diarrhea in Bangladesh where it was associated to a 74% reduced risk of xerophthalmia [67]. In contrast, a study in China found breastfeeding as a risk factor of VAD among 0–5 year-old children when SR was used as an indicator [68]. Breast milk provides a reliable source of highly bioavailable VA and is a large contributor to VA requirements among children in developing countries. Its contribution ranged from 42% to 56% and from 33% to 61% of the VA requirements among 6–12 and 12–23 month-old African children, respectively, filling the gap of VA intakes from the diet [69–73]. This result calls for action to reinforce the advocacy for optimal breastfeeding practices up to and beyond 2 years as a prevention strategy of VAD while improving VA intakes from the diet. This is urgent, given the decrease of the median breastfeeding duration (18 months) observed in our study compared to the previously observed time in Dakar (21 months) [32].

Finally, even though VALS of children who received at least one VA supplement since birth were similar to that of those who never received any VA supplements in the non-VAS group based on MRDR values, the number of VA supplements consumed since birth might have contributed to children’s VALS. This was observed in the South African study where a positive trend was found between the number of VA supplements and children’s total liver reserves measured using RID [58]. This is possible, given that administration of VA supplements is part of the protocols for the clinical management of childhood illnesses such as persistent diarrhea, measles and severe acute malnutrition among children under five in Senegal [74,75]. However, data regarding the history of VA supplement administration beyond the 6 months preceding the study was not collected to support this hypothesis.

### High prevalence of infections and inflammation

The high prevalence of infections and subclinical inflammation observed in this study was actually of concern as immunization coverage in this part of the country is of utmost importance [32,76]. Children’s environmental health in Dakar may have deteriorated in recent years as witnessed by poor air quality, inappropriate waste management and repeated floods during wet season [77–80], explaining the difference with previously reported figures (65.5%). In this situation, morbidity prevention and control through the mitigation of environmental risks should be a priority intervention.

However, the extent of infections and inflammation, which appears comparable between the 2 groups, do not presuppose an effectiveness of VAS on the occurrence of infections 2 to 6 months post-VAS. This is consistent with the results of the Ghana VAS Trials (VAST) study that reported no effect of VAS on the occurrence of diarrhea and acute respiratory infections among children 6 to 90 months old at 4 months post-VAS [81]. Also, the meta-analysis of Grotto and colleagues found no effect of VAS on diarrhea occurrence but an increase in the occurrence of respiratory tract infections [82]. In a recent systematic review and meta-analysis, no difference was observed in the incidence of diarrhea or lower respiratory tract infections among children supplemented with VA compared to those who were not [83]. This might be linked to the fact that VAS acts by decreasing the severity but not necessarily the incidence of infections as formerly postulated [84].

### Do we consider scaling-back VAS in this subpopulation?

Using the GAVA decision-making framework [27], the virtual absence of VAD in this urban population of children and the suggested adequacy of VA dietary intakes stated above, question the necessity to pursue universal VAS in Senegal. To consider a targeted VAS implementation scheme by scaling-back VAS in this subpopulation while closely monitoring its impact should be examined, because of the potential risk of VA excess due to concurrent substantial intakes of preformed VA through fortified oil, VA supplements as well as VA from breastmilk and other dietary origin. For instance, van Stuijvenberg and colleagues detected a high rate of hypervitaminotic liver reserves among South African children exposed to VA fortification and VAS from a community where liver is frequently eaten and where they previously found a 5.8% prevalence of VAD, similar to ours [58,85]. This is congruent with the very high proportion of children identified as non-deficient in VA by both indicators. According to Tanumihardjo, liver reserves might be substantial in these cases [86]. Unfortunately, the indicators used herein, MRDR and SR, are not useful in determining hypervitaminotic to toxic VA status [86,87]. Thus, these issues need further investigation using more sensitive methods like the RID technique.

### Correlation between MRDR values and SR concentrations

In our study, a significant correlation between the MRDR values and SR was noticed as found in Indonesian women [88] by contrast with Senegalese, Ghanaian and Indonesian children in previous studies [54,55,89]. MRDR values and SR have been correlated when the latter were very low or very high [86]. In this study, we found a median SR value of 1.3 μmol/L, higher than 1.05 μmol/L, the threshold usually applied for vitamin A insufficiency [86], which means that at least 50% of children had high SR. Moreover, almost 12% were above 1.6 μmol/L, an area of agreement between SR and the MRDR value [86]. These results suggest that SR of the majority of the children (62%) were high and might explain this relationship.

### MRDR is more informative than SR concentration alone

Our findings reiterate the fact that SR is not a suitable indicator of VA status alone at the individual level. Firstly, nearly 5% (n = 11) of children were false positive, i.e., presented adequate SR whereas their VALS (DR:R ≥ 0.060) were deficient, suggesting an influence of recent VA dietary intake. The second fact is that SR was depressed by subclinical inflammation. This was confirmed by the negative significant correlation observed between SR and inflammation biomarkers, which does not exist using the MRDR values. Thus, this is a confirmation that the MRDR value, which is not influenced by inflammation, is a more accurate indicator of VA status of individuals than SR.

## Study limitations

The retrospective assessment of exposure has limitations, although several precautions have been taken to minimize their impact. Whether or not the child was supplemented was assessed in three stages: 1) reviewing the health facility's supplementation records to determine whether or not each child was supplemented and to record the date of last supplementation for those who were supplemented at least once, 2) checking and comparing the supplementation status and date of latest supplementation recorded in the health card with that of the health post, and 3) interviewing the mother to determine whether the child may have received another vitamin A supplement not recorded in the health card. Eligible children are those for whom all these stages have been completed. However, it is still possible that there may have been an error in the reporting of information at the health posts, or that a child received a vitamin A supplement at another health facility without this being noted in the health card and without the mother remembering, thus introducing an information bias with possible errors in the classification of subjects within the two groups. In addition, the children recruited for this study are followed up in health facilities where their mothers and/or caregivers can access nutritional counseling and adequate care. Therefore, they may be less likely to be vitamin A deficient compared to those who do not attend the health services for diverse reasons. This approach was made to minimize biases related to VAS status that might have been greater without cross-checking health facility records and health cards.

## Conclusion

Using the MRDR test, this study did not find an enhancement of VALS or a reduction in VAD among VAS children compared to their non-supplemented counterparts, 2 to 6 months after VA administration. In this population, VALS are adequate and dietary intakes appear to be enough, probably due to synergistic actions of diverse interventions/practices. Reinforcing breastfeeding and strengthening morbidity prevention and control are essential components in consolidating these achievements. The virtual elimination of VAD as well as the existing risk of hypervitaminosis A calls to consider a targeted VAS implementation scheme in Senegal, implying the scaling back of VAS in this subpopulation. However, gathering quantitative evidence of sufficient dietary VA intakes and conducting more sensitive assessment of liver stores using isotopic techniques should be considered in this population to better guide policy and programmatic decision-making of VAS.

## Supporting information

S1 File (PDF)

S2 File (PDF)
