# Supplementary material for: Adequate vitamin A liver stores estimated by the modified relative dose response test are positively associated with breastfeeding but not vitamin A supplementation in Senegalese urban children 9–23 months old: A comparative cross-sectional study
Source: PLoS One. 2021 Jan 29;16(1):e0246246. doi: 10.1371/journal.pone.0246246 (PMC7846024; doi:10.1371/journal.pone.0246246)
Supplement: S1 File — (PDF) [file pone.0246246.s001.pdf]

REPUBLIQUE DU SENEGAL
Un Peuple – Un But – Une Foi

MINISTERE DE LA SANTE
ET DE L'ACTION SOCIALE

NERS
SENEGAL
Comité National d'Ethique
pour la Recherche en Santé

N° 0 0 0 0 0 164 MSAS/DPRS/CNERS

Dakar, le 0 6 SEP 2019

Le Président

AVIS ETHIQUE ET SCIENTIFIQUE

***Référence :* Protocole SEN19/54 : « Impact de la supplémentation en vitamine A sur le statut et les réserves hépatiques en vitamine A d'enfants âgés de 6-23 mois à Dakar à l'aide du test MRDR ».**

Professeur,

J'accuse réception de vos réponses aux questions relatives au protocole en référence ci-dessus. À l'analyse, le Comité National d'Ethique pour la Recherche en Santé les trouve globalement satisfaisantes. En conséquence, le comité émet un avis éthique et scientifique favorable pour permettre la mise en œuvre dudit protocole.

Cet avis a une durée d'une année à compter de sa date de signature. Son renouvellement reste assujetti à la présentation d'un rapport d'étape permettant d'être informé sur le niveau de mise en œuvre de l'étude.

Je vous prie de croire, ***Professeur***, à l'assurance de ma considération distinguée et de mes encouragements renouvelés.

Pr. Anta Tal DIA
COMITÉ NATIONAL D'ÉTHIQUE POUR LA RECHERCHE EN SANTÉ
La Présidente

Pr. Nicole Idohou-Dossou
FST/UCAD
Chercheur Principal de l'Etude

Ministère de la Santé et de l'Action Sociale ; Direction de la Planification, de la Recherche et des Statistiques ; Division de la Recherche : tel 77 361 42 12 email : cnrs2008@live.fr; N° Assurance du CNERS : IORG0002135 valable jusqu'au 28 février 2022.
