# Supplementary material for: Adequate vitamin A liver stores estimated by the modified relative dose response test are positively associated with breastfeeding but not vitamin A supplementation in Senegalese urban children 9–23 months old: A comparative cross-sectional study
Source: PLoS One. 2021 Jan 29;16(1):e0246246. doi: 10.1371/journal.pone.0246246 (PMC7846024; doi:10.1371/journal.pone.0246246)
Supplement: S2 File — (PDF) [file pone.0246246.s002.pdf]

Université Cheikh Anta Diop de Dakar

Comité d'Ethique de la Recherche (CER)

Dakar le 08 juillet 2019

A Mme Mane Hélène FAYE
Etudiante à l'Ecole doctorale: "Sciences de la vie, de la santé et de l'environnement",
UCAD, Dakar, Sénégal

Référence : Protocole 0397/2019/CER/UCAD : "Impact de la supplémentation en vitamine A sur le statut et les réserves hépatiques en vitamine A d'enfants âgés de 6 à 23 mois à Dakar à l'aide du test MRDR."

Approbation éthique de la recherche

Votre protocole a été examiné selon les règles édictées par le Comité National d'Ethique pour la Recherche en Santé (CNERS) du Sénégal et conformément aux procédures établies par l'Université Cheikh Anta DIOP de Dakar (UCAD) pour l'approbation éthique de toute recherche impliquant des participants humains.

J'ai le plaisir de vous informer que, sur la base des informations fournies dans le protocole, le Comité d'Ethique de la Recherche (CER) de l'UCAD considère que la recherche proposée, respecte les normes éthiques appropriées et en conséquence, approuve son exécution.

Le CER attire votre attention sur tout changement ultérieur dans la recherche qui soulèverait des questions éthiques non incluses dans le protocole original. Ces changements devront être soumis au Comité d'Ethique de la Recherche pour approbation.

Le Président,
Professeur Alioune DIEYE

COMITE D'ETHIQUE DE LA RECHERCHE
Université Cheikh Anta DIOP de Dakar
Le Président

Siège : Faculté de Médecine, de Pharmacie et d'Odontologie – FMPO
Ancien Bureau de l'Ecole Doctorale « SEV » à côté du Nouveau Bâtiment
B.P.5005, Dakar, Sénégal. Téléphone : +221 77 467 77 93
Email : comite.ethique@ucad.edu.sn
